# Gene expression following induction of regeneration in *Drosophila *wing imaginal discs. Expression profile of regenerating wing discs

**DOI:** 10.1186/1471-213X-10-94

**Published:** 2010-09-02

**Authors:** Enrique Blanco, Marina Ruiz-Romero, Sergi Beltran, Manel Bosch, Adrià Punset, Florenci Serras, Montserrat Corominas

**Affiliations:** 1 Serveis Científico-Tècnics de la Universitat de Barcelona (SCT-UB), Barcelona, Catalonia, Spain; 2 Fibran SA, Sant Joan de les Abadesses, Catalonia, Spain; 3 Departament de Genètica, and Institut de Biomedicina de la Universitat de Barcelona (IBUB), Diagonal 645, 08028 Barcelona, Catalonia, Spain

## Abstract

**Background:**

Regeneration is the ability of an organism to rebuild a body part that has been damaged or amputated, and can be studied at the molecular level using model organisms. *Drosophila *imaginal discs, which are the larval primordia of adult cuticular structures, are capable of undergoing regenerative growth after transplantation and *in vivo *culture into the adult abdomen.

**Results:**

Using expression profile analyses, we studied the regenerative behaviour of wing discs at 0, 24 and 72 hours after fragmentation and implantation into adult females. Based on expression level, we generated a catalogue of genes with putative role in wing disc regeneration, identifying four classes: 1) genes with differential expression within the first 24 hours; 2) genes with differential expression between 24 and 72 hours; 3) genes that changed significantly in expression levels between the two time periods; 4) genes with a sustained increase or decrease in their expression levels throughout regeneration. Among these genes, we identified members of the JNK and Notch signalling pathways and chromatin regulators. Through computational analysis, we recognized putative binding sites for transcription factors downstream of these pathways that are conserved in multiple *Drosophilids*, indicating a potential relationship between members of the different gene classes. Experimental data from genetic mutants provide evidence of a requirement of selected genes in wing disc regeneration.

**Conclusions:**

We have been able to distinguish various classes of genes involved in early and late steps of the regeneration process. Our data suggests the integration of signalling pathways in the promoters of regulated genes.

## Background

The process of regeneration allows organisms to recreate the original shape, size and function of body parts that have been lost or damaged. Regenerative capacity varies between species, ranging from simple wound healing to unrestricted regeneration of all body parts [1,2]. Since the basis of regeneration was first established by T. H. Morgan [3], an extensive body of literature has been published describing the different mechanisms of regeneration employed in many different species. For instance, regeneration of complete individuals from any tiny body fragment has been studied in freshwater planarians and hydra [4,5], and limb regeneration after amputation has been explored in detail in amphibians and teleost fish [6-8]. Regeneration of heart, liver, pancreas, and other organs has been also extensively studied in zebrafish, mouse and human [9-12].

*Drosophila *imaginal discs, the larval primordia of adult cuticular structures, are capable of undergoing regenerative growth. When imaginal discs are manually fragmented and cultured in the abdomen of adult flies, cells at the wound site undergo proliferation and regenerate the missing part. Pioneering experiments demonstrated that regeneration induces limited cell plasticity, enabling the reconstitution of missing tissue while disc identity is maintained (reviewed in [13,14]). In rare cases, however, the initial fate was lost in some subsets of proliferating cells, which acquired the identity of another disc type in a phenomenon named transdetermination [15,16]. As in many other systems, regeneration of imaginal discs involves wound healing and blastema formation [17,18]. In a rapid response to injury, epithelial and cytoskeletal changes occur during the first 24 hours. Concomitantly, local proliferation increases and peaks around 2-3 days after fragmentation [19,20]. This in vivo culture system has proven to be a powerful method for studying the regenerative process at the tissue and cellular level, and what is known about the underlying molecular mechanisms implicates several signalling pathways.

The JNK signal transduction cascade is activated by exposure of cells to cytokines or environmental stress. Multiple studies have demonstrated that JNK regulates cell proliferation, apoptosis, inflammatory responses, tissue morphogenesis, and polarity [21,22]. In the *Drosophila *embryo, several downstream target genes of this signalling pathway are involved in dorsal closure and thorax formation [23,24]. The JNK pathway is required during imaginal disc regeneration [17,18,25] and is activated near the wound as well as in cell death-induced regeneration [26]. The Wnt signalling cascade plays a key role in most aspects of embryonic development [27] and is involved in multiple processes during regeneration [28,29]. Induction of ectopic expression of *wingless *(*wg)*, a member of the Wnt family, mimics the pattern changes observed in leg imaginal discs after fragmentation (including regeneration), and promotes cell-fate plasticity such as leg-to-wing transdetermination [30,31]. The Notch signalling pathway is essential to determine cell fate and regulate pattern formation during embryonic and adult life [32]. It has been also reported to participate in the regeneration of zebrafish heart [33], *Xenopus *tail [6], mice muscle [34] and in transdetermination of imaginal discs [31]. Finally, *dpp *is induced by the JNK pathway in the leading edge cells during dorsal closure [22] although no upregulation of its expression has been found during the process of regeneration [35].

The outcome of these signalling pathways is the transcriptional regulation of target genes that will elicit the ultimate response. Precisely which genes are required for the process of regeneration has been examined in the last few years by the use of genetic and molecular techniques in various model organisms (such as planarians, hydra, amphibians and zebrafish). For example, a large-scale RNAi-based screen was performed to survey gene function in planarian regeneration [36] and a global analysis of gene expression was carried out in *Xenopus *limb regeneration [37]. In *Drosophila*, a collection of lethal P-lacZ enhancer trap lines was used to screen for genes that function in leg disc regeneration [20]. Klebes et al. [31] reported the expression profiles of cells induced by ectopic *wg *expression to transdetermine from leg to wing disc, thus generating a list of candidate regulators of cellular plasticity in flies. Despite these studies, however, it remains unclear whether regeneration requires the reactivation of earlier developmental genes or signalling pathways, or if it involves the activation of novel genes specific to the regeneration process. In an attempt to answer these questions we have taken a systematic approach and determined the expression profile of regenerating wing imaginal discs at different times after fragmentation and culture. By combining experimental and computational techniques, we have been able to decipher the transcriptional status of regenerating discs and link signalling circuits to gene regulation.

## Results and Discussion

### Whole genome expression analysis of intact and regenerating wing discs

Previous studies from our group showed that epithelial and cytoskeleton changes occur during the first 24 hours of regeneration [17] and that proliferation peaks two to three days after the cut [19]. To study different stages of the regenerative process we designed 12 microarrays containing 12,254 genes annotated in RefSeq from *D. melanogaster *[38]. Four microarrays (non cut, NC0→NC24) were used to assess the effect of the implantation procedure in intact wing discs. The remaining eight were used to measure changes in gene expression in the first 24 hours after disc dissection and implantation (cut, C0→C24) and during the period between 24 hours and 72 hours after the cut (C24→C72). The entire set of microarrays was normalized following the same protocol, extracting in each case the list of significant genes (at least two-fold change, false discovery rate (FDR)-corrected *P *value < 0.05, see Methods). The genes identified in these microarrays were functionally annotated using the Gene Ontology (GO terms [39]).

The number of genes whose expression was significantly modified during regeneration is shown in Table 1. More genes were reported in C0→C24 in comparison to NC0→NC24, which reflects the combination of regeneration events and the implantation effect at this early stage. In fact, half of the genes whose expression was significantly upregulated or downregulated during this period in cut discs were not detected in non-fragmented ones (44% of 1,183 differentially expressed genes in C0→C24 were not found in NC0→NC24). Conversely, most relevant genes in the intact discs transcriptome presented the same expression pattern in cut discs (87% of 763 genes in NC0→NC24 were in C0→C24). The number of genes in C0→C24 was also higher than in C24→C72 confirming the strong initial response during the first 24 hours.

**Table 1:** Total number of up and downregulated genes in NC0→NC24, C0→C24 and C24→C72 microarrays.

	NC0→NC24	C0→C24	C24→C72
**Genes ↑**	407	607	116

**Genes ↓**	356	576	165

**TOTAL**	763	1183	281

**Figure 1:**
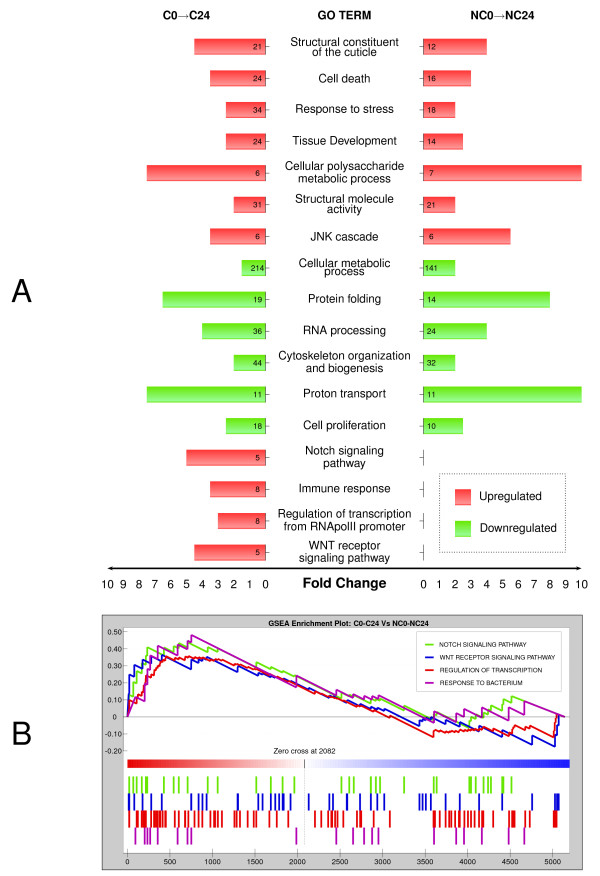
**Whole genome expression analysis of cut (C) and uncut (NC) wing imaginal discs after 24 hours**. (A) Gene ontology (GO) terms of upregulated (red) or downregulated (green) genes. The number of genes in each category in the microarrays is shown within the bars. The length of the bars indicates the fold change (enrichment in these transcriptomes compared to the whole genome, *P *value < 0.001 in all cases). (B) Enrichment plots for statistically significant GO categories. (Top) The Enrichment Score (ES) computed by GSEA is shown for each category. ES value reaches its absolute peak on the left side of the enrichment plot, indicating overrepresentation in C0→C24 compared with NC0→NC24. The zero cross mark indicates the point in which the difference between expression in C0→C24 and NC0→NC24 is 0. (Bottom) Coloured bars illustrate the position of genes belonging to each GO category ranked according to their expression values in C and NC discs.

Functional annotation of both C0→C24 and NC0→NC24 microarrays reveals significant enrichment in genes associated with apoptosis, response to stress, cytoskeletal activity, and JNK pathway regulation (Figure 1A), which agrees well with previous results reported for early regeneration of wing imaginal discs [17,18]. The cellular machinery required for gene expression (RNA processing and protein synthesis) seems to be blocked during the first 24 hours after implantation. We analyzed the set of genes displaying expression changes only in cut and implanted discs (200 upregulated and 220 downregulated genes in C0→C24), which presumably represents the early regeneration signature in wing imaginal discs. Many upregulated genes (Figure 1A) are associated with the immune response to other organisms and probably constitute a response to surgical manipulation. It has been reported that mechanical wounding is able to induce an antibacterial response that might prime the organism to fight what is perceived to be an increased likelihood of infection [40]. More importantly, we identified several genes involved in the Notch and Wg signalling pathways and transcription factor-encoding genes whose expression is increased only in cut discs during the first 24 hours (Additional file 1). Functional analysis of downregulated genes identified enrichment associated with multiple metabolic processes.

While direct comparison between upregulated and downregulated genes in C0→C24 and NC0→NC24 provides a qualitative description of both transcriptomes, GSEA (Gene Set Enrichment Analysis) is able to reconstruct a quantitative portrait of the functional differences between these microarrays. GSEA is a computational method that determines whether a defined set of genes (e.g. GO categories) shows statistically significant differences between two biological conditions (e.g. cut versus intact discs) [41]. Genes associated with a given GO category were ranked according to their expression profiles (C0→C24 versus NC0→NC24). Then, the enrichment score (ES) was calculated to evaluate if the GO terms were randomly distributed or found at the extremes (left or right) of the ranked list. While GSEA analysis detected a significant enrichment in C0→C24 (*P *value < 0.01) of genes involved in Notch and Wg signalling pathways, several transcription factors and the immune response (Figure 1B), no particular categories were found to be specific only in intact discs. This observation strengthens the early regeneration signature identified by direct comparisons of upregulated and downregulated genes.

By comparing the differential gene expression between cut and uncut discs, we have been able to describe the implantation effect at the transcriptomic level. However, as biological processes governing disc regeneration may be pertinent for the implantation response, a clear distinction between C0→C24 and NC0→NC24 gene sets is rather difficult to make at this point. For instance, genes involved in apoptosis and regulation of the JNK cascade, which have been reported to be essential for imaginal disc wound healing and dorsal closure [17,24], were identified as upregulated in microarrays for intact discs. Implantation probably results in sufficient stress to trigger the JNK pathway and these genes cannot be eliminated as relevant. Moreover, members of the Notch and Wg signalling pathways, which show increased expression only in cut discs, have been previously reported in other regeneration systems [31,33]. As an alternative, the nonsurgical method for inducing tissue damage and regeneration [26,28] emerges as a very powerful system not only to avoid the technical difficulties associated with disc transplantation but also to perform new molecular screens and validate our results.

### Identification of genes with putative roles in regeneration

**Figure 2:**
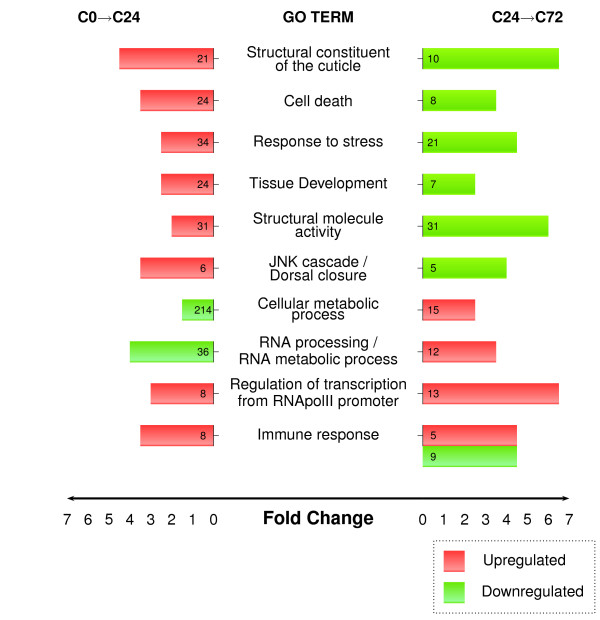
**Functional annotation of differentially expressed genes in wing imaginal discs at 24 and 72 hours of regeneration**. Gene ontology annotation of upregulated (red) or downregulated (green) genes. The number of genes in each category in the microarrays is shown within the bars. The length of the bars indicates the fold change (enrichment in these transcriptomes compared to the whole genome, *P *value < 0.001 in all cases).

We next examined the set of 281 genes showing expression changes in the C24→C72 microarrays and detected an enrichment of transcription factors during this second regeneration stage (Figure 2 and Additional file 2). When compared to C0→C24 experiments, we observed a significant increase in genes involved in the regulation of RNA metabolism and gene expression in the set of upregulated genes, whereas genes involved in apoptotic processes, structural activities and dorsal closure were augmented in the set of downregulated genes (Figure 2). These results suggest that the normal activity of imaginal discs, interrupted in response to dissection and implantation, is resumed during the 24-72 hours of regeneration. In addition, we detected functional categories associated with the immune system in both, upregulated genes (related to the stress response) and downregulated genes (related to the defense response to bacteria).

**Figure 3:**
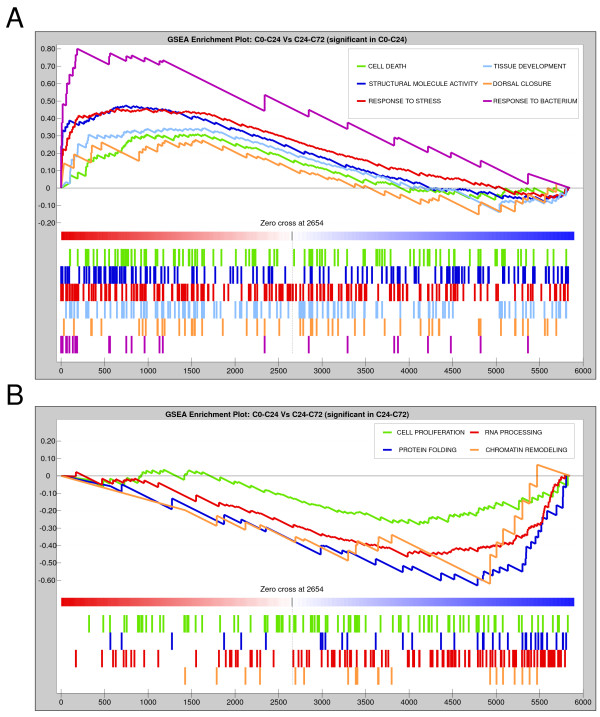
**Gene set enrichment analysis of differentially expressed genes in wing imaginal discs at 24 and 72 hours of regeneration**. (A) Enrichment plots for statistically significant GO categories in C0→C24. ES value reaches its absolute peak on the left side of the enrichment plot, indicating overrepresentation of these categories compared with C24→C72. (B) Enrichment plots for statistically significant GO categories in C24→C72. ES value reaches its absolute peak on the right side of the enrichment plot, indicating overrepresentation of these categories compared with C0→C24. Genes in each GO category ranked according to their expression values in both time points are shown as coloured bars. The zero cross mark indicates the point in which the difference between expression in C0→C24 and C24→C72 is 0.

We performed GSEA analysis of C0→C24 and C24→C72 microarrays in order to elucidate which GO categories are enriched in the full transcriptomes. The GSEA plot in Figure 3A shows the functional classes overrepresented in early regeneration. The results of that analysis confirmed the enrichments previously identified (Figure 2). Moreover, in addition to RNA processing and protein folding activities, GSEA analysis of C24→C72 identified an enrichment in genes associated with cell proliferation and chromatin remodeling processes during late regeneration of wing discs (Figure 3B).

**Figure 4:**
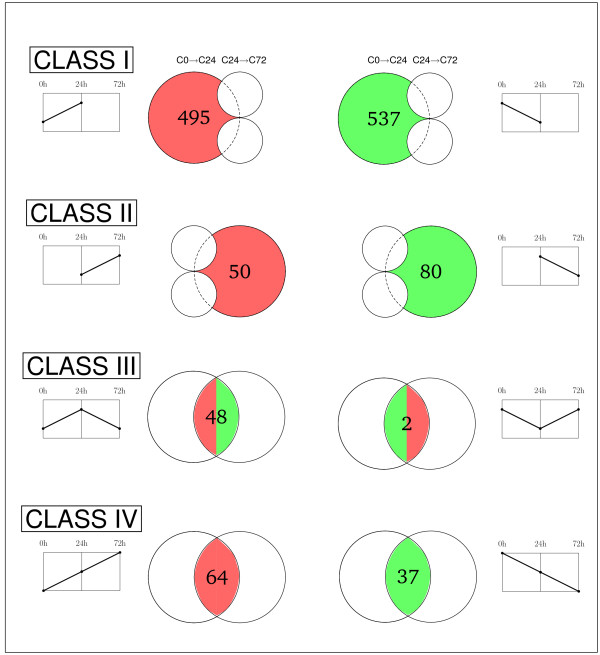
**Classification of differentially expressed genes involved in wing imaginal disc regeneration**. According to the expression changes in early and late regeneration stages, genes in the transcriptomes were organized in four classes: Class I, genes with differential expression only in the first 24 hours; Class II, genes with differential expression only between 24 and 72 hours; Class III, genes with a significant difference between the two time periods; Class IV, genes with a sustained increase or decrease in their expression levels during regeneration. Intersections of upregulated genes (in red) and downregulated genes (in green) in a particular class are depicted using Venn diagrams. For each intersection, we display the number of genes and a generic pictogram representing the gene expression trend in C0→C24 and C24→C72.

**Figure 5:**
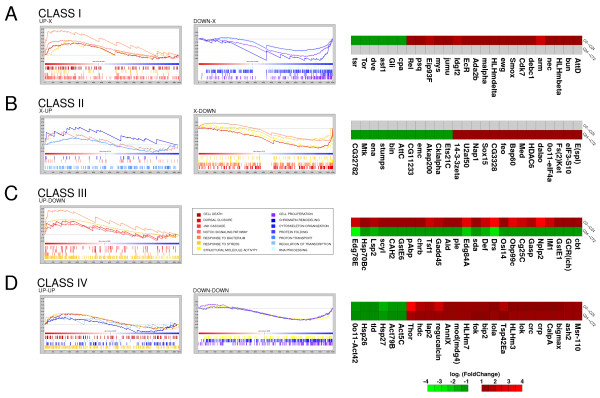
**Gene class signature of wing imaginal disc regeneration**. Functional signature and expression map for each gene class defined during wing imaginal disc regeneration. (Left panel) Enrichment plots for statistically significant GO categories identified for Class I (A), Class II (B), Class III (C) and Class IV (D) genes. ES values indicate overrepresentation of GO terms in each class. The zero cross mark indicates the point in which the ratio between expression in C0→C24 and C24→C72 is 0. (Right panel) Expression maps of representative genes of each class. Red is used for upregulation, green for downregulation and grey for no significant change in expression levels (see scale bar).

To further characterize the regeneration process, we defined four classes of genes according to their expression levels (Figure 4): Class I, genes showing differential expression only in C0→C24; Class II, genes with differential expression only in C24→C72; Class III, genes displaying changing expression levels between the two periods; and Class IV, genes that steadily increase or decrease their expression levels. For each class, we graphically defined their characteristic functional signature using GSEA analysis (for a list of representative genes, see Figure 5 and Additional file 3).

Class I genes show a significant change, either increasing or decreasing expression between 0 hours and 24 hours after the cut, but remain constant during the second period of time. Most genes in C0→C24 present this expression pattern (82% of upregulated genes and 93% of downregulated genes, Additional file 4). As expected, we found upregulated genes associated with dorsal closure, the JNK cascade, MAP kinase activity, and the Notch and Wg signalling pathways. In addition, other genes associated with imaginal disc development, immune response, and apoptotic processes were detected. Moreover, we identified several downregulated genes in this class associated with growth regulation or involved in chromatin remodeling and wing disc development. This category is a representation of the additive response of the implantation effect and the process of regeneration.

Class II genes display increased or decreased expression between 24 and 72 hours but remain constant in C0→C24. Approximately half of the genes in C24→C72 showed this expression pattern (44% of upregulated genes and 49% of downregulated genes, Additional file 4). A significant enrichment of upregulated transcription factors was observed in Class II, including *Sox box protein 15 (Sox15 or SoxF*), *Enhancer of split (E(spl)) *and *Medea (Med)*. *SoxF *codes for a transcription factor involved in the Wg signalling pathway that has been linked to control of proliferation in *Drosophila *[42] and also skeletal muscle regeneration in mice [43]. *E(spl) *is an essential Notch signalling pathway mediator [32] and *Med*, a component of the *dpp *pathway [44]. Moreover, several chromatin regulators showing increasing expression levels are also included in this class. Brahma associated protein 60 kD (Bap60) and Dalao are components of the Brahma complex involved in chromatin remodeling [45] and Nucleosome assembly protein 1 (Nap1) has been implicated in nucleosome assembly [46]. *Bap60 *and *Sox15 *have been also identified in microarrays of leg disc transdetermination [31]. Activation of these genes together with the presence in this class of splicing and translation initiation factors indicates that the normal RNA processing machinery resumes its activity in the disc at this stage. In contrast, genes involved in the wound healing response and cytoskeletal organization processes were downregulated, presumably indicating that cell shape changes and cytoskeletal reorganization described in early healing have been accomplished.

Class III represents the set of genes whose expression changed dramatically, from significant upregulation to downregulation or vice versa. Up to 48 genes were identified as upregulated in C0→C24 and downregulated in C24→C72 (29% of downregulated genes in C24→C72, Additional file 4) but only two genes had the opposite expression pattern. This module represented only 8% of upregulated genes in C0→C24 (Additional file 4). Class III was, in fact, enriched in genes associated with the stress response, response to stimuli, defense response, and structural activities, as well as several downstream targets of the JNK regulatory cascade. For example, we found the Krüppel-like transcription factor *cabut *(*cbt*) [47,48], the *Collagen type IV *(*Cg25C*) gene related to dorsal closure, and *Drosomycin *(*Drs*), *Immune induced molecule I *(*IM1*), *Transferrin *(*Tsf1*) and *Gadd45*, which play a role in the defensive response [40,49]. The increase in the expression of other genes that play a defensive function during the first 24 hours and the subsequent decrease up to 72 hours correlates well with their role in the defensive immune response. Furthermore, the *slamdance *(*sda*) gene, belonging to this category, has also been identified in other regeneration screens using leg imaginal discs [31]. Most genes identified in Class III exhibit an increase/decrease pattern of expression during regeneration. These genes could account for the cellular responses to injury, which would then be switched off once wound healing is completed.

Finally, Class IV includes genes whose expression remains significantly increased or decreased throughout the whole process, indicating their relevance during the 72 hours after the cut. A large fraction of upregulated and downregulated genes in C24→C72 (56% of upregulated genes and 22% of downregulated genes, Additional file 4) had the same expression pattern observed in C0→C24. While both microarrays were characterized by an enrichment of upregulated genes whose products are involved in apoptotic processes and transcription factors, the set of downregulated genes was rich in products with defensive response functions. Among the genes whose expression pattern was upregulated throughout the experiment, we found *headcase *(*hdc*) and *regucalcin*, which were previously identified in imaginal disc regeneration [31]. In addition, we detected the *cryptocephal *(*crc) *gene, different chromatin remodeling factors such as *absent, small, or homeotic discs 2 *(*ash2*) and *modifier of mdg4 *(*mod(mdg4)*) as well as three basic helix-loop-helix (bHLH) transcription factors (*bigmax*, *HLHm3 *and *HLHm7*), indicating again that transcriptional regulation plays a critical role in regeneration. Finally, *Inhibitor of apoptosis 2 *(*Iap2*), *longitudinals lacking *(*lola*), and *Thor *are related to the immune response. *Crc *is a downstream target of the JNK pathway implicated in wound healing [22,50] and it has been reported that the activity of *Thor *in aging also depends on the JNK pathway [51]. The set of genes that remained downregulated throughout the 72-hour period comprised a group of actin and heat shock proteins that were probably activated just after the injury, and the endopeptidases *tolloid *(*tld*) and *tolkin *(*tok*), involved in imaginal disc morphogenesis.

### Transcriptional regulators acting in early and late regeneration

Among the plethora of genes identified as having altered expression during the regeneration process, we draw attention to the potential role played by those associated with transcriptional regulation. We first analyzed the putative targets of several transcription factors which are candidate participants in disc wound healing and regeneration. We computationally searched for binding sites of these transcription factors in promoter sequences of misregulated genes, using the genomes of 12 *Drosophilas *[52] to solidify the predictions (conserved sites at least in *D. pseudoobscura *and four additional *Drosophilids*, enrichment calculated in comparison to the total number of conserved sites of each class in the *D. melanogaster *genome, see Methods).

**Figure 6:**
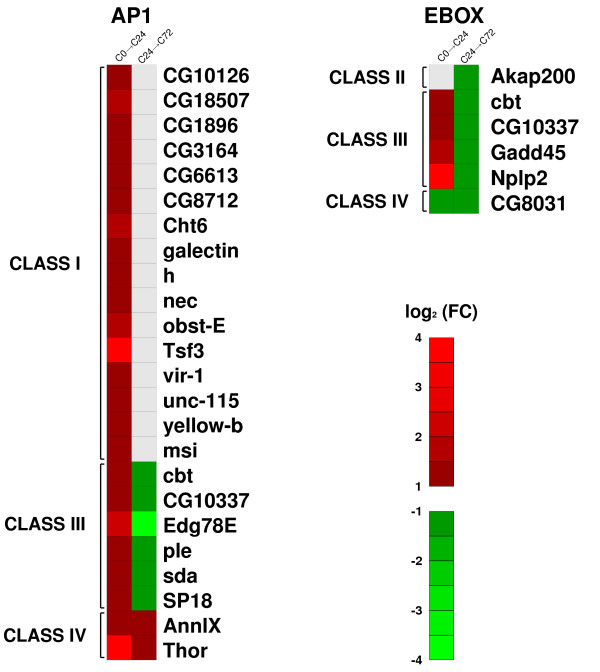
**Genes containing putative transcription factor binding sites**. Expression maps of genes of different classes containing putative consensus sites for AP1 and E-boxes. For each microarray (C0→C24 and C24→C72), upregulated genes are shown in red, downregulated genes in green and genes with no differential expression in grey. On the left, genes in C0→C24 with AP1 binding sites in the promoter region. On the right, genes in C24→C72 containing E-boxes.

The JNK signalling pathway activates the AP1 (Activator Protein 1) transcription factor, a dimer of *jun *and *fos *[53,54]. We searched for AP1 binding sites in the promoters of upregulated and downregulated genes in C0→C24 (Figure 6). We found putative binding sites in the promoters of 24 upregulated genes conserved in several *Drosophila *species (371 genes identified in the whole genome, *P *value < 10^-6^). Interestingly, 11 out of these 24 genes were reported only in cut discs. The number of AP1 predictions in the downregulated genes was not statistically significant. This was as anticipated, given the role of AP1 as a transcriptional activator. The number of AP1 occurrences was not significant in the upregulated genes of the C24→C72 microarrays, while 15 AP1 binding sites were identified in 12 downregulated genes (*P *value < 10^-8^). When correlating these predictions with the gene classes previously established, in addition to the expected abundance of Class I genes, we identified a significant enrichment of Class III genes. Despite a small amount of misregulated genes belong to this class (Additional file 4), we identified AP1 sites in six Class III genes in both C0→C24 and C24→C72 microarrays (*P *value < 0.001 and *P *value < 0.05, respectively). These results suggest that the JNK pathway regulates the expression of Class I and Class III genes through AP1 during the first few hours of wing disc healing and that its activity decreases during later stages of regeneration. As expected, different elements of the JNK pathway have been identified in our expression profiles, confirming its activation during regeneration. The phosphatase *puckered *(*puc*) has been used as a molecular readout of the activated JNK pathway and its expression seems directly controlled by AP1. In imaginal disc fragmentation experiments, the expression of *puc *is activated in several rows of cells near the wound edges at 5 hours after the fragmentation, peaking at 12 hours and decreasing from 24 hours onwards, as the wound is healed [17,19]. Differences in *puc *expression would therefore be very difficult to detect at the time points used in this study. However, by using bioinformatics analysis we have identified a significant enrichment of AP1 sites in the promoters of several other genes with differential expression only in cut discs that are pyhlogenetically conserved in multiple *Drosophila *species, suggesting that they could be direct targets of the JNK pathway.

Many members of the (*E(spl)*) gene complex show a significant increase in their expression levels during wound healing and regeneration stages. In particular, we observed that the *E(spl) *gene is upregulated in C24→C72 microarrays (Class II). *E(spl) *is a bHLH transcription factor that binds regulatory sequences containing the E-box palindromic motif CACGTG [55,56]. We performed a search of E-boxes in the promoters of misregulated genes in C24→C72 (Figure 6). We identified six evolutionarily conserved E-boxes in the promoters of downregulated genes (346 genes in the whole genome of *D. melanogaster*, *P *value < 0.01). Four of those six genes display an expression pattern fitting Class III genes (*P *value < 0.05), in contrast to the lower total number of genes in this class in C24→C72 (Additional file 4). Although these results suggest that the genes identified are potential downstream targets of the Notch pathway, it should be pointed out that other proteins, such as dMyc, could also recognize the general consensus sequence for the E-box element [57].

Finally, besides transcription factors we have also identified genes that encode for chromatin remodelers. This finding highlights the importance of transcription and chromatin remodeling in regeneration and is consistent with similar studies [31]. It has been demonstrated that suppression of Polycomb group (PcG) proteins by JNK induces transdetermination in *Drosophila *imaginal discs and that this downregulation is directly controlled by the JNK signalling pathway [25]. We have not found PcG genes in our screen. Instead, the majority of chromatin regulators encode proteins that may play a general role as transcriptional activators. Among these, Ash2, a member of the *trithorax *group (trxG), is required for histone H3 trimethylation at lysine 4 (H3K4me3) and belongs to multiple methylation complexes [58,59], and BAP60 and Dalao are members of the Brahma complex [60,61]. The transcriptional activation of this small number of cofactors may lead to the enzymatic activation of several proteins involved in chromatin activity. According to our results, global transcription slows at the beginning of regeneration but resumes concurrent to wound repair.

### Requirement of transcription factors and chromatin remodelers in regeneration

**Figure 7:**
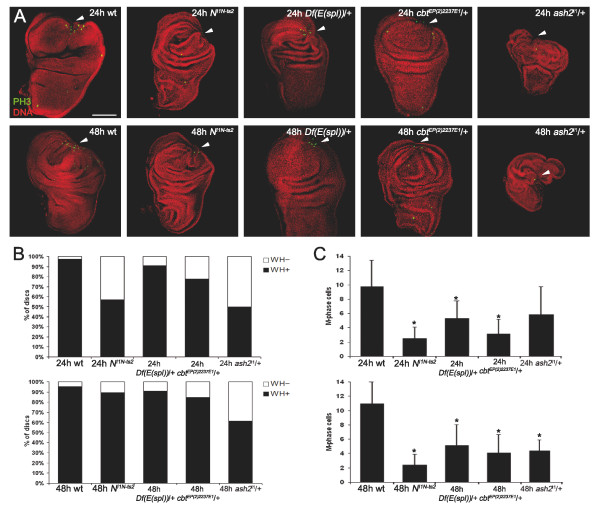
**Involvement of the Notch pathway, *cbt *and *ash2 *in wing imaginal disc regeneration**. Wound healing (WH) and mitosis (M) in blastemas of Notch pathway, *cbt *and *ash2 *mutants. (A) Regenerating imaginal discs from wild-type (wt); *N*^*I1N-ts2*^; Df(*E(spl)*)/+; *cbt*^*EP(2)2237E1*^/+; and *ash2*^*I1*^/+. Staining of mitosis (green) and nuclei (red). Scale bar = 100 μm. Upper panels show 24-hour regenerating discs; lower panels, 48-hour regenerating discs. Arrowheads point to the wound vertex. (B) Percentage of discs with correct wound closure (black) and absence of closure (white). (C) Number of M-Phase cells in the blastema region at 24 and 48 hours. Asterisk indicates differences between wt and mutants discs (*P *value < 0.005).

We expect impairment on the ability to regenerate in mutants for the genes identified in our molecular screen. The requirement of the JNK pathway in wing imaginal disc regeneration has already been described [17-19,26] and alterations in the expression levels of Notch members have also been reported [31]. After validating the changes in expression levels of selected genes by quantitative PCR (Additional file 5), we investigated their involvement in regeneration. Wing discs from heterozygous flies (the homozygous condition being lethal) were fragmented as above, implanted and recovered after 24 and 48 hours (Figure 7). Although healing did not seem to be affected in *N*^*I1N-ts2 *^mutant discs, proliferation, measured by counting the number of mitotic cells labelled with anti-Phospho-Histone3 (PH3), was impaired. We also analyzed regeneration in imaginal discs from heterozygous flies with a deficiency of all *E(spl) *complex genes. Despite the fact that wound closure did not seem to be compromised, a significant decrease in proliferation occurs at 24 and 48 hours. Moreover, to inquire into the role of Class III genes in regeneration, we examined *cbt *mutant discs. Even though the discs healed properly, there was lower proliferation in analyzed discs. Finally, since the category of chromatin regulators is significant (this report and [31]), we examined the requirement for *ash2*, a Class IV member, in regeneration. Heterozygous discs for the *ash2*^*I1 *^allele were smaller and showed wound healing defects at 24 hours, probably hampering the proper assessment of their proliferative capacity. Although proliferation seems to be affected at 48 hours, it is probable that the absence of regeneration in these mutant discs is due to healing impairment.

### The transcription factor Cbt as an example of Class III genes

**Figure 8:**
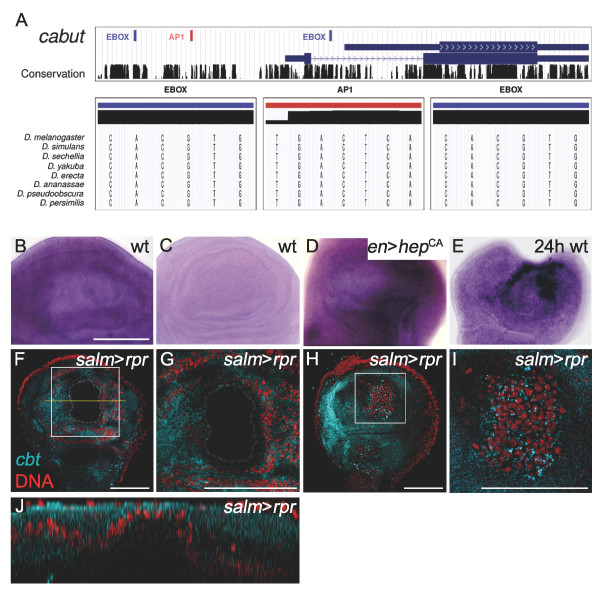
**Analysis of *cbt *promoter, expression and requirement in cell death- induced regeneration**. (A) Graphical representation of one AP1 site and two E-boxes that are evolutionarily conserved in the promoter of *cbt *and detailed sequences of each binding site in different *Drosophila *species. This picture was produced using the UCSC genome browser [76]. (B-E) In situ hybridizations in wing imaginal discs. (B) Wild-type disc showing ubiquitous *cbt *expression. (C) Wild-type disc with *cbt *sense RNA probe as a control. (D) *cbt *expression in *en *>*hep*^CA ^disc. Notice upregulation of *cbt *in the posterior compartment. (E) Confocal section of a wing disc after 24 hours of regeneration. *cbt *is overexpressed near the wound. (F-J) Fluorescent in situ hybridization (blue) in disc in which cell death has been induced; nuclei are stained red. (F) Basal confocal section of a wing disc after 10 hours of *rpr *induction in the *salm *domain. The dotted line indicates the cell death domain. *cbt *expression is constant throughout the disc. (G) High-magnification view of the white square in F. (H) Apical confocal section of a wing disc after 10 hours of *rpr *induction in the *salm *domain. Regenerating cells in this domain show a high level of *cbt *expression. (I) High-magnification view of the white square in H, showing increased expression of *cbt*. (J) Cross-section through a stack of images (from apical to basal) at the level of the yellow line in F. Scale bars = 50 μm.

Most genes identified in Class III display a characteristic increase/decrease pattern of expression during regeneration, suggestive of tight regulation associated with the requirement of the proteins encoded by these genes in a particular window of time. As a member of Class III, *cbt *was upregulated during the first 24 hours after disc fragmentation, decreasing dramatically in the following 48-hour period. As already suggested by Muñoz *et al. *[47], we found an AP1 binding site present in the proximal promoter of *cbt *conserved in multiple *Drosophila *species (Figure 8A), which indicates that is directly regulated by AP1. In third instar larvae, *cbt *is ubiquitously expressed in the wing disc (Figure 8B, C), and according to our predictions, we observed an increase in the level of expression of *cbt *after activating the JNK pathway in the posterior compartment (Figure 8D). More importantly, we detected an increase in the regenerating tissue, confirming the induction of its expression after injury (Figure 8E). As an alternative method to avoid microsurgery, regeneration was induced by triggering apoptosis in the wing disc in a spatially and temporally regulated manner. Recent reports have already shown that cell death can be locally induced in certain domains of the disc using the Gal4/UAS binary system in combination with Gal80^ts ^[26,28]. The use of the *salm-Gal4 *construct to drive expression of the pro-apoptotic gene *reaper *(*rpr*) results in a region of dead cells in the *spalt (sal) *domain. Higher levels of *cbt *expression can be detected in the regenerating cells that close the wound apically (Figure 8E-H). We have already demonstrated that during cell death-induced regeneration, the JNK pathway is activated at the leading edges of healing tissue, and is required in the living cells for the regulation of healing and regenerative growth [26]. Our results point to the transcription factor Cbt as a crucial downstream mediator gene of JNK signalling during microsurgery or cell death-induced regeneration.

Although further experiments are required, it is possible that E(spl) binds to the E-boxes identified in the promoters of *cbt *and other members of Class III genes contributing to their downregulation in the 24-72 hours period. In fact, in addition to *cbt *and *CG10337*, we found three more genes of this class (*Cg25C, Gadd45 *and *ple*) containing conserved AP1 sites and E-boxes in their promoter regions when we extended our analysis up to 10 Kb upstream of the TSS (Additional file 6). All these genes are precisely known JNK targets [40,47,62,63]. In *Drosophila*, the Notch pathway is known to participate in the regulation of growth in the wing [64] and a relationship between both the JNK and Notch pathways has recently been documented in tissue homeostasis in aging flies [65]. In that study, it was shown that tissue regeneration in the fly intestinal epithelium depends on a sensitive balance between JNK and Notch signalling events regulating stress responses, stem cell proliferation, and cell differentiation. Therefore, it is tempting to speculate that both JNK and Notch pathways cooperate by regulating the transcriptional activity of the same set of genes during wound healing and regeneration of wing imaginal discs.

## Conclusions

By determining expression profiles at different times of regeneration, we have been able to identify early and late genes involved in the process. The onset of wound healing is the first necessary step for regeneration [66] and the role of the JNK pathway in this type of processes has been widely documented [17,19,25]. Different elements of the JNK pathway have been identified in our expression profiles, confirming its activation during regeneration. Our analysis show a significant enrichment of AP1 sites in the promoters of several genes with differential expression only in cut discs, suggesting that they could be direct targets of the JNK pathway. Several genes identified in our work encode for transcription factors, some of them of known signalling pathways, and chromatin remodelers. This finding highlights the importance of transcription and chromatin dynamics in regeneration and is consistent with similar studies [31]. A comprehensive description of the regeneration process will be enriched in the future by incorporating information complementary to our expression data. Thus, additional biological processes that are not directed by transcriptional responses, such as translational control and subcellular localization, should be recognized. However, the characterization of the relative contribution of critical pathways [67], or more precisely, of key genes may ultimately lead to the identification of therapeutic targets for use in regenerative medicine.

## Methods

### *Drosophila *strains and experimental conditions

All *Drosophila *strains and crosses were kept on standard media at 25°C. For microarray and qRT-PCR experiments, imaginal disc regeneration was induced in the *w*^1118^ISO; 2iso; 3iso isogenic line from the DrosDel collection [68]. The following strains were used: CS; *ash2*^*I1*^/TM6C [69]; *N*^*I1N-ts2*^rb'; FRT82*gro*^+^: Df(3R)*gro*^b32.2^/TM6B (a complete deficiency of all *E(spl) *complex genes, in this paper referred to as Df(*E(spl)*); *cbt*^*EP(2)2237E1*^/CyO-twi:GFP [47]; *en*-Gal4;Gal80^ts^/SM6a-TM6B; UAS-*hep*^CA^. All strains were kept at a constant temperature of 25°C, with the exception of *Notch*^*I1N-ts2*^, which was kept at 17°C until the dissection of discs and then at 25°C from just after implantation until the end of the experiment.

For cell death induction, UAS-*rpr*/Gal80^ts^; *salm*-Gal4 flies were used as described [26]. Larvae were kept at 17°C to avoid *rpr *expression until third instar larvae (approximately 120 hours after egg laying). Next, they were shifted to 29°C to activate *rpr *expression for 10 hours, and then larvae were dissected and fixed with 4% formaldehyde.

### Imaginal disc manipulation and labeling of mitosis

Imaginal disc manipulation, either of wild type or mutant discs, was performed as described previously [17]. Wing discs were removed from third instar larvae and a 90° sector was dissected out from the posterior (P) compartment, leaving a 3/4 anterior fragment. Experimental and control (uncut) discs were implanted into recently eclosed Canton S females and kept at 25°C. Regenerating fragments were recovered at 24 and 48 hours after implantation, fixed with 4% paraformaldehyd and immunostained following standard protocols with anti-PH3 (1:1000, Upstate Biotechnology, Inc), FITC-conjugated goat anti-rabbit secondary antibody (1: 200, Jackson Immunoresearch, Inc.) and TOPRO3 (Molecular Probes, Inc) for nuclei staining. Imaginal discs were mounted in SlowFade Light Antifade (Molecular Probes, Inc.) prior to confocal analyses (Leica SPE). Images were treated with ImageJ (NIH) and Adobe Photoshop software. For analysis of wound healing and proliferation, at least 10 discs were analyzed for each condition. The number of M-phase cells near the wound was determined using ImageJ (NIH) software. We used SPSS Statistics 17.0 for statistical analysis.

### In situ hybridization

In situ hybridization of wing imaginal discs fixed with 4% formaldehyde was performed as described previously [70]. *cbt *sense and antisense RNA probes were synthesized using a complete cDNA (a gift from N. Paricio) with DIG RNA labeling Mix (Roche, Inc.) and hydrolyzed prior to hybridization. An antibody conjugated with alkaline phosphatase (Roche, Inc.) was used against Digoxigenin probes. NBT/BCIP (Roche, Inc.) was used to develop in situ hybridizations and fast red tablets (Roche, Inc.) for fluorescent in situ hybridization. Discs were analyzed with a Leica DMLB fluorescent microscope and a Leica SP2 confocal microscope.

### Quantitative RT-PCR analysis

Total RNA was extracted using the Mini RNA Isolation I Kit™(Zymo Research Corp., CA, USA). Reverse transcription reactions with 500 ng of RNA isolated from regenerating discs were used to synthesize cDNA with M-MLV reverse transcriptase (Invitrogen Corp., Carlsbad, CA, USA) according to the manufacturer's instructions. The qRT-PCR was performed with an ABI PRISM 7700 following the recommended protocol (Applied Biosystems, Foster City, CA, USA). Each sample was replicated three times and average values were used for further analysis. Data were analyzed by the ΔCT method and normalized by subtracting the value of the geometric average of three control genes (*dia*, *mRpL9 *and *ptp61F*) obtained using geNorm software [71]. TaqMan primers and probes designed and synthesized by Applied Biosystems for this analysis were: Dm02150755_g1 (*ash2*); Dm01800197_s1 (*cbt*); Dm02151501_s1 (*E(spl*)); Dm01821420_m1 (*Sox15*); Dm02151361_g1 (*tld*), Dm01811206_g1 (*dia*); Dm02135860_s1 (*mRpL9*); Dm 0183210_g1 (*ptp61F*).

### Microarray analysis

Microarrays were printed at the Plataforma de Transcriptòmica (SCT-PCB, Universitat de Barcelona, Spain) using the *Drosophila *genome Oligo Set version 1.1 (Operon Biotechnologies Inc., Huntsville, AL., USA) as described previously [69,72] (GEO platform number GPL3797). Total RNA was extracted as described above from wing discs recovered after 0, 24 and 72 hours for cut discs, and at 0 and 24 hours for uncut discs. At least two independent RNA extractions were carried out. In the case of cut discs, RNA extracted after 24 hours of implantation was used as a common reference and therefore three pair-wise comparisons were set: C0→C24, C24→C72 and NC0→NC24. Four microarrays were hybridized for each experiment in biological replicate pairs including dye swaps to take dye bias into account. Microarray analyses were performed as described previously [72]. GenePix Results (GPR) data files were obtained for each microarray with an Axon 4000B scanner and GenePix Pro 6 (Molecular Devices Corp., Sunnyvale, CA, USA). All GPR files were analyzed with the Limma package from BioConductor [73,74] using the same criteria. Array normalization was carried out independently for each set of four arrays using the *mad *method from OLIN, and a linear model was fitted and FDR corrected [73]. We obtained a list of genes that displayed at least two-fold differential expression (FDR-corrected *P *value < 0.05). Further experimental and statistical details together with raw and normalized data can be accessed through NCBI's Gene Expression Omnibus [75] series number GSE17408.

All gene sets have been mapped in the genome of *D. melanogaster *using the RefSeq track of the UCSC genome browser [76] annotations (genome assembly dm3, April 2006). We used the TermEnrichment tool from the AmiGO suite [77] to assess the statistical significance of GO term enrichments on each list of genes using the whole genome as a second reference set (*P *value < 0.001, each enrichment must contain at least five genes). We employed the program GSEA [41] to perform gene-set-enrichment analyses in the full transcriptome of each microarray (rank function: difference of expression values). The Enrichment Score (ES) was calculated by walking down the ranked list, increasing the cumulative sum when a gene is present in a given GO category and decreasing it if a gene is not (see [41] for further details).

### Promoter characterization

We extracted 1,000 nucleotides upstream of the transcription start site of each gene according to RefSeq annotations in the UCSC Genome browser [76]. Using the predictive models published in the literature for AP1 and E(spl) [54,55], we used the MatScan program [78] to obtain the list of putative transcription factor binding sites on the set of gene promoters. We converted these predictions into the UCSC custom track format to map them along the *D. melanogaster *genome. Using the Conservation track (multiple alignment of *Drosophila *species), we filtered out the predictions that were not conserved in at least five species (including *D. pseudoobscura *or more distant species). We randomly sampled 10,000 datasets containing the same number of genes of each up or downregulated gene sets, using a Z-test to evaluate the statistical significance on each set of predictions in comparison to the whole genome.

## Authors' contributions

EB performed the bioinformatics analysis; SB, MB, FS and MC conceived and designed the experiments; MRR, SB, MB and AP performed the experiments; FS and MC contributed reagents and materials; EB and MC wrote the paper. All authors read and approved the final manuscript.

## Supplementary Material

Additional file 1 **Gene signature of wing imaginal disc early regeneration**. For each gene we display the functional annotation according to the Gene Ontology. Click here for file

Additional file 2 **List of transcription factors in C24→C72**. For each transcription factor we display the binding molecule (DNA or protein) and the functional annotation according to the Gene Ontology. Click here for file

Additional file 3 **Representatives of regeneration gene classes**. For each class in the catalogue we show the list of selected representatives and the functional annotation according to the Gene Ontology. Click here for file

Additional file 4 **Number of genes on each class in C0→C24 and C24→C72**. For each microarray we display the number of misregulated genes distributed in classes according to the gene catalogue. Click here for file

Additional file 5 **Quantitative RT-PCR for target genes in regenerating discs after 0, 24 and 72 hours**. Twenty-four hour expression levels were used as reference for comparison (baseline). Arrows represent significant expression changes (*P *< 0.005); red arrows indicate upregulation and green arrows downregulation. Click here for file

Additional file 6 **AP1 sites and E-boxes identified in the promoter region of Class III genes**. For each gene we display the length of the promoter sequence, the position of AP1 sites (in red) and E-boxes (in blue), the beginning of the gene (in white) and the conservation level of the sequence according to the multiple alignment of *Drosophila *species (UCSC genome browser Conservation track). Click here for file
