# Supplementary material for: Gene expression following induction of regeneration in Drosophila wing imaginal discs. Expression profile of regenerating wing discs
Source: BMC Dev Biol. 2010 Sep 2;10:94. doi: 10.1186/1471-213X-10-94 (PMC2939566; doi:10.1186/1471-213X-10-94)
Supplement: Additional file 1 — Gene signature of wing imaginal disc early regeneration. For each gene we display the functional annotation according to the Gene Ontology. [file 1471-213X-10-94-S1.PDF]

C0→C24 ↑ (only)				
**Gene**	**GO annotation**	**log FC(C0→C24)**	***P* value**	**log FC(NC0→NC24)**
arm	transcription activator activity, protein binding, protein binding, cell adhesion, cell morphogenesis, dorsal closure, imaginal disc-derived wing expansion, positive regulation of JNK cascade, wing disc morphogenesis	2.28	10^{-11}	0.14
bun	cell fate determination, RNA polymerase II transcription factor activity, decapentaplegic receptor signaling pathway, negative regulation of cell fate specification, negative regulation of Notch signaling pathway	0.78	10^{-2}	0.50
Cbl	negative regulation of epidermal growth factor receptor activity, imaginal disc-derived wing morphogenesis, dorsal/ventral axis specification	0.76	10^{-2}	0.50
EcR	transcription factor activity, sequence-specific DNA binding, ecdysteroid hormone receptor activity, steroid binding, autophagy, imaginal disc-derived wing morphogenesis, metamorphosis, epidermis development	1.11	10^{-4}	0.00
Fps85D	dorsal closure, elongation of leading edge cells, photoreceptor cell morphogenesis, protein amino acid phosphorylation, actin cable formation	0.62	10^{-2}	0.00
HLHm7	transcription factor activity, compound eye development, dendrite morphogenesis	0.64	10^{-2}	0.29
HLHmbeta	transcription factor activity, Notch signaling pathway, negative regulation of transcription from RNA polymerase II promoter, DNA binding	0.77	10^{-2}	-0.05
HLHmdelta	transcription factor activity, Notch signaling pathway, negative regulation of transcription from RNA polymerase II promoter, DNA binding	0.63	10^{-2}	0.19
Idgf2	imaginal disc growth factor activity, imaginal disc development, nutrient reservoir activity	1.93	10^{-8}	0.00
nec	Toll signaling pathway, antifungal humoral response, immune response	0.99	10^{-3}	0.31
Mpk2	MAPKKK cascade, MAP kinase activity defense response, immune response	0.64	10^{-2}	0.56
psq	transcription factor activity, regulation of transcription, DNA-dependent, imaginal disc-derived wing morphogenesis	0.97	10^{-3}	0.00

Additional file 1.

Gene signature of wing imaginal disc early regeneration
