# Supplementary material for: Gene expression following induction of regeneration in Drosophila wing imaginal discs. Expression profile of regenerating wing discs
Source: BMC Dev Biol. 2010 Sep 2;10:94. doi: 10.1186/1471-213X-10-94 (PMC2939566; doi:10.1186/1471-213X-10-94)
Supplement: Additional file 2 — List of transcription factors in C24→C72. For each transcription factor we display the binding molecule (DNA or protein) and the functional annotation according to the Gene Ontology. [file 1471-213X-10-94-S2.PDF]

C24→C72 ↑					
**Gene**	**Gene name**	**Binding**	**GO terms**	**log FC(C24→C72)**	***P* value**
Bap60	Brahma associated protein 60kD	Protein	brahma complex chromatin , remodeling complex, dendrite morphogenesis, muscle development	0.79	10^{-3}
bigmax	bigmax	DNA	transcription factor activity, autophagic cell death, dendrite morphogenesis, muscle development	0.61	10^{-2}
bip2	bip2	Protein	transcription factor TFIID complex	0.90	10^{-4}
crc	cryptocephal	DNA	metamorphosis pupation	0.63	10^{-3}
crp	cropped	Protein	transcription regulator activity	0.66	10^{-3}
dalao	dalao	Protein	chromatin remodeling, brahma complex	0.68	10^{-3}
E(spl)	Enhancer of split	DNA	transcription repressor activity, Notch signaling pathway, cell fate commitment, dendrite morphogenesis, epidermal growth factor receptor, imaginal disc-derived wing margin morphogenesis	1.04	10^{-5}
HLHm3	E(spl) region transcript m3	DNA	specific transcriptional repressor activity, Notch signaling pathway	0.70	10^{-3}
HLHm7	E(spl) region transcript m7	DNA	compound eye development, dendrite morphogenesis	0.69	10^{-3}
lola	longitudinals lacking	Protein	structural molecule activity, antimicrobial humoral response, axonogenesis	0.59	10^{-2}
Med	Medea	DNA	BMP signaling pathway, cell proliferation, imaginal disc-derived wing morphogenesis, transforming growth factor beta receptor, somatic stem cell maintenance	0.62	10^{-2}
mod(mdg4)	modifier of mdg4	Protein	chromatin binding, establishment or maintenance of chromatin architecture, induction of apoptosis	0.68	10^{-3}
Sox15	Sox box protein 15	DNA	ATP binding, aminoacyl-tRNA ligase activity	0.65	10^{-3}
**C24→C72 ↓**					
**Gene**	**Gene name**	**Binding**	**GO terms**	**log FC(C24→C72)**	***P* value**
bin	Biniou	DNA	mesodermal cell fate commitment salivary gland morphogenesis	-0.80	10^{-3}
Ets21C	Ets at 21C	DNA	dendrite morphogenesis	-0.82	10^{-4}

Additional file 2.

List of transcription factors in C24→C72
