# Supplementary material for: Gene expression following induction of regeneration in Drosophila wing imaginal discs. Expression profile of regenerating wing discs
Source: BMC Dev Biol. 2010 Sep 2;10:94. doi: 10.1186/1471-213X-10-94 (PMC2939566; doi:10.1186/1471-213X-10-94)
Supplement: Additional file 3 — Representatives of regeneration gene classes. For each class in the catalogue we show the list of selected representatives and the functional annotation according to the Gene Ontology. [file 1471-213X-10-94-S3.PDF]

CLASS I					
**Genes ↑ -**	**GO terms**	**log FC(C0→C24)**	***P* value**	**log FC(C24→C72)**	***P* value**
Ada2b	chromatin remodeling, positive regulation of histone acetylation, SAGA complex, DNA binding	1.01	10^{-3}	-0.04	NS
arm	transcription activator activity, protein binding, protein binding, cell adhesion, cell morphogenesis, dorsal closure, imaginal disc-derived wing expansion, positive regulation of JNK cascade, wing disc morphogenesis	2.28	10^{-11}	0.00	NS
Atg6	autophagic cell death, salivary gland cell autophagic cell death	0.68	10^{-2}	0.38	NS
B4	imaginal disc development, circadian rhythm	0.97	10^{-3}	0.37	NS
bun	RNA polymerase II transcription factor activity, autophagic cell death, negative regulation of Notch signaling pathway, decapentaplegic receptor signaling pathway	0.78	10^{-2}	0.40	NS
crq	scavenger receptor activity, apoptosis, autophagic cell death, immune response, phagocytosis, engulfment, membrane	1.39	10^{-5}	-0.30	NS
Cdk7	general RNA polymerase II transcription factor activity, protein serine/threonine kinase activity	0.88	10^{-3}	0.23	NS
CG16708	activation of protein kinase C activity by G-protein coupled receptor protein signaling pathway, autophagic cell death	0.97	10^{-3}	0.55	NS
dap	cyclin-dependent protein kinase inhibitor activity, negative regulation of cell cycle	0.84	10^{-3}	0.23	NS
debcl	induction of apoptosis, DNA damage response, signal transduction resulting in induction of apoptosis, negative regulation of neuron apoptosis	1.15	10^{-4}	0.39	NS
dos	antimicrobial humoral response, determination of anterior/posterior axis, embryo, regulation of Ras protein signal transduction, torso signaling pathway	0.66	10^{-2}	0.30	NS
Dpt	antibacterial humoral response, defense response to bacterium, innate immune response	1.17	10^{-4}	0.00	NS
EcR	transcription factor activity, sequence-specific DNA binding, ecdysteroid hormone receptor activity, steroid binding, autophagy, imaginal disc-derived wing morphogenesis, metamorphosis, epidermis development	1.11	10^{-4}	-0.02	NS
Eip93F	transcription factor activity,	1.95	10^{-8}	0.28	NS

	autophagic cell death, induction of apoptosis by hormones, phagocytosis, engulfment,				
ewg	specific RNA polymerase II transcription factor activity, imaginal disc morphogenesis, DNA binding, central nervous system development, muscle development	0.85	10^{-3}	0.43	NS
Fps85D	protein binding, dorsal closure, dorsal closure, elongation of leading edge cells, adherens junction, membrane	0.62	10^{-2}	0.00	NS
HLHmbeta	transcription factor activity, Notch signaling pathway, negative regulation of transcription from RNA polymerase II promoter, DNA binding	0.77	10^{-2}	-0.38	NS
HLHmdelta	transcription factor activity, Notch signaling pathway, negative regulation of transcription from RNA polymerase II promoter, DNA binding	0.62	10^{-2}	0.27	NS
Idgf2	imaginal disc development, imaginal disc growth factor activity	1.93	10^{-8}	-0.03	NS
jumu	chromatin binding, transcription factor activity, sequence-specific DNA binding, dorsal closure, establishment or maintenance of chromatin architecture, imaginal disc-derived wing morphogenesis	1.32	10^{-4}	0.01	NS
malpha	Notch signaling pathway, cell fate specification	0.75	10^{-2}	0.52	NS
Mef2	RNA polymerase II transcription factor activity, sequence-specific DNA binding, muscle fiber development, skeletal muscle development	1.14	10^{-4}	0.00	NS
Mpk2	MAP kinase activity, MAPKKK cascade, immune response, response to stress	0.64	10^{-2}	-0.15	NS
mys	cell adhesion molecule binding, apposition of dorsal and ventral imaginal disc-derived wing surfaces, cell migration, cell-matrix adhesion, dorsal closure, regulation of cell shape, maintenance of epithelial integrity, open tracheal system	0.69	10^{-2}	-0.09	NS
nec	Toll signaling pathway, antifungal humoral response, immune response	0.99	10^{-3}	-0.27	NS
stck	apposition of dorsal and ventral imaginal disc-derived wing surfaces, dorsal closure, embryonic pattern specification, negative regulation of JNK cascade, cell leading edge	0.96	10^{-3}	0.58	NS
PGRP-SA	Toll signaling pathway, detection of bacterium, immune response, pattern recognition receptor activity	1.38	10^{-5}	-0.53	NS

Phk-3	Ras protein signal transduction, response to bacterium, torso signaling pathway	1.89	10^{-8}	-0.40	NS
psq	transcription factor activity, imaginal disc-derived wing morphogenesis, DNA binding, protein binding	0.97	10^{-3}	0.10	NS
Rel	transcription factor activity, Toll signaling pathway, immune response, positive regulation of transcription from RNA polymerase II promoter, response to bacterium	1.35	10^{-5}	0.10	NS
Smox	transcription factor activity, transforming growth factor beta receptor signaling pathway, imaginal disc-derived wing morphogenesis	0.69	10^{-2}	0.19	NS
trbl	protein kinase activity, negative regulation of mitosis	1.18	10^{-4}	0.45	NS
unk	imaginal disc-derived wing morphogenesis, DNA binding, bristle morphogenesis	1.34	10^{-5}	0.19	NS
**Genes ↓ -**	**GO terms**	**log FC(C0→C24)**	***P* value**	**log FC(C24→C72)**	***P* value**
asf1	establishment or maintenance of chromatin architecture, nucleosome assembly, regulation of cell cycle, regulation of nuclear mRNA splicing, via spliceosome	-1.05	10^{-3}	-0.32	NS
blw	growth, ATP synthesis coupled proton transport, phagocytosis, engulfment	-0.84	10^{-3}	0.14	NS
bonsai	regulation of growth, translation, structural constituent of ribosome	-0.59	10^{-2}	0.00	NS
cpa	Wing disc development, actin cytoskeleton organization	-0.65	10^{-2}	-0.29	NS
dom	establishment or maintenance of chromatin architecture, cell proliferation, regulation of alternative nuclear mRNA splicing, via spliceosome, gene silencing, chromatin remodeling complex	-0.60	10^{-2}	0.04	NS
dve	imaginal disc-derived wing morphogenesis, transcription factor activity	-0.75	10^{-2}	-0.21	NS
Gli	maintenance of imaginal disc-derived wing hair orientation	-0.60	10^{-2}	-0.28	NS
path	regulation of growth, amino acid transport	-1.31	10^{-5}	0.00	NS
Rpd3	histone deacetylase activity, chromatin silencing, gene silencing	-0.87	10^{-3}	-0.43	NS
Samuel	regulation of growth, spermatogenesis	-0.62	10^{-2}	0.00	NS
Tor	positive regulation of cell growth, positive regulation of cell size, determination of adult life span	-0.69	10^{-2}	-0.11	NS
tsr	actin binding, establishment of imaginal disc-derived wing hair	-0.91	10^{-3}	-0.17	NS

	orientation				
**CLASS II**					
**Genes -↑**	**GO terms**	**log FC(C0→C24)**	***P* value**	**log FC(C24→C72)**	***P* value**
Bap60	Brahma complex, chromatin remodeling, general RNA polymerase II transcription factor activity	0.54	NS	0.79	10^{-5}
CG3328	transcription factor activity, caspase activity	0.55	NS	0.76	10^{-4}
dalao	Brahma complex, chromatin remodeling, general RNA polymerase II transcription factor activity	0.18	NS	0.68	10^{-4}
eIF3-S10	translation initiation factor activity	0.23	NS	0.62	10^{-3}
eIF-4a	translation initiation factor activity, regulation of alternative nuclear mRNA splicing, via spliceosome, imaginal disc growth, dorsal/ventral axis specification	0.18	NS	0.68	10^{-4}
E(spl)	transcription factor activity, transcription repressor activity, Notch signaling pathway, cell fate commitment, dendrite morphogenesis, epidermal growth factor receptor signaling pathway, imaginal disc-derived wing margin morphogenesis	-0.03	NS	1.04	10^{-7}
feo	cytokinesis, mitotic spindle stabilization	0.48	NS	0.72	10^{-4}
Fs(2)Ket	regulation of cell shape, actin filament organization,	0.32	NS	0.82	10^{-5}
HDAC6	histone deacetylation	0.23	NS	0.79	10^{-5}
Med	RNA polymerase II transcription factor activity, BMP signaling pathway, cell proliferation, imaginal disc-derived wing morphogenesis, transforming growth factor beta receptor signaling pathway	0.11	NS	0.62	10^{-3}
Nap1	nucleosome assembly, regulation of transcription, DNA-dependent, histone binding	0.57	NS	1.08	10^{-7}
Sox15	transcription factor activity, ATP binding, aminoacyl-tRNA ligase activity	0.48	NS	0.65	10^{-4}
U2af50	spliceosome assembly, nuclear mRNA splicing, via spliceosome, RNA splicing factor activity, transesterification mechanism	0.50	NS	0.91	10^{-6}
**Genes -↓**	**GO terms**	**log FC(C0→C24)**	***P* value**	**log FC(C24→C72)**	***P* value**
14-3-3zeta	protein binding, Ras protein signal transduction, cell proliferation, protein kinase C inhibitor activity	-0.34	NS	-0.62	10^{-3}
Akap200	negative regulation of Ras protein signal transduction, autophagic cell death, protein kinase A binding	-0.31	NS	-0.71	10^{-4}

AttC	defense response to bacterium, antibacterial humoral response	0.44	NS	-0.70	10^{-4}
bin	transcription factor activity, sequence-specific DNA binding, mesodermal cell fate commitment, salivary gland morphogenesis	0.50	NS	-0.80	10^{-5}
emc	negative regulation of transcription from RNA polymerase II promoter, protein binding, dorsal closure, imaginal disc-derived wing morphogenesis, cell proliferation	-0.24	NS	-0.66	10^{-4}
ena	dorsal closure, cytoskeleton organization and biogenesis, regulation of cell shape	0.15	NS	-0.65	10^{-4}
Ets21C	transcription factor activity, sequence-specific DNA binding, dendrite morphogenesis	0.00	NS	-0.82	10^{-5}
L(1)19Ec	immune response, dorsal closure, central nervous system development, peripheral nervous system development	-0.14	NS	-0.61	10^{-3}
Mtk	antibacterial humoral response, antifungal humoral response	0.00	NS	-1.16	10^{-8}
stumps	epithelial cell migration, open tracheal system, mesodermal cell migration fibroblast growth factor receptor signaling pathway	0.36	NS	-0.76	10^{-4}
tlk	protein kinase activity, establishment and/or maintenance of chromatin architecture, regulation of cell shape, histone phosphorylation, antimicrobial humoral response	0.11	NS	-0.67	10^{-4}
**CLASS III**					
**Genes ↑ ↓**	**GO terms**	**log FC(C0→C24)**	***P* value**	**log FC(C24→C72)**	***P* value**
cbt	JNK cascade, dorsal closure, transcription activator activity, positive regulation of transcription, autophagic cell death	1.25	10^{-4}	-0.64	10^{-4}
Cg25C	extracellular matrix structural constituent, collagen type IV, dorsal closure	2.02	10^{-9}	-0.68	10^{-4}
chrb	cell death, negative regulation of signal transduction, negative regulation of growth	0.64	10^{-2}	-0.71	10^{-4}
Drs	antibacterial humoral response, antifungal humoral response, innate immune response	4.43	0	-3.11	0
Gadd45	JNK cascade	1.82	10^{-7}	-1.12	10^{-8}
IM1	defense response, extracellular region	2.16	10^{-10}	-0.94	10^{-6}
scyl	cell death, negative regulation of growth, negative regulation of signal transduction	0.79	10^{-2}	-0.80	10^{-5}
sda	response to mechanical stimulus, mechanosensory behavior	0.79	10^{-2}	-0.60	10^{-3}

Tsf1	defense response, cellular iron ion homeostasis	2.41	10^{-12}	-1.41	10^{-12}
**CLASS IV**					
**Genes ↑↑**	**GO terms**	**log FC(C0→C24)**	***P* value**	**log FC(C24→C72)**	***P* value**
ash2	protein binding, imaginal disc-derived wing morphogenesis, chromatin-mediated maintenance of transcription	1.01	10^{-3}	0.78	10^{-5}
bigmax	transcription factor activity, autophagic cell death, dendrite morphogenesis	0.80	10^{-2}	0.61	10^{-3}
bip2	regulation of transcription from RNA polymerase II promoter, protein binding, transcription factor TFIID complex	0.60	10^{-2}	0.90	10^{-6}
crc	transcription factor activity, metamorphosis, pupation, sequence-specific DNA binding	0.78	10^{-2}	0.63	10^{-3}
crp	RNA polymerase II transcription factor activity	0.80	10^{-2}	0.66	10^{-4}
hdc	cell differentiation, open tracheal system development	1.85	10^{-8}	0.75	10^{-4}
HLHm3	transcription factor activity, specific transcriptional repressor activity, Notch signaling pathway	0.98	10^{-3}	0.70	10^{-4}
HLHm7	transcription factor activity, compound eye development, dendrite morphogenesis	0.64	10^{-2}	0.69	10^{-4}
Iap2	anti-apoptosis, antimicrobial humoral response	1.30	10^{-4}	0.69	10^{-4}
lok	protein kinase activity, DNA damage response, signal transduction resulting in induction of apoptosis	0.82	10^{-2}	0.66	10^{-4}
mod(mdg4)	RNA polymerase II transcription factor activity, chromatin binding, establishment or maintenance of chromatin architecture, induction of apoptosis	0.61	10^{-2}	0.68	10^{-4}
regucalcin	lipid particle	2.04	10^{-9}	0.63	10^{-3}
thor	regulation of cell growth, negative regulation of cell size, immune response, antibacterial humoral response	3.13	0	1.44	10^{-12}
**Genes ↓↓**	**GO terms**	**log FC(C0→C24)**	***P* value**	**log FC(C24→C72)**	***P* value**
Act42A	structural constituent of cytoskeleton, cytoskeleton organization and biogenesis, phagocytosis, engulfment	-0.75	10^{-2}	-0.87	10^{-5}
Act5C	structural constituent of cytoskeleton, cytoskeleton organization and biogenesis, phagocytosis, engulfment	-0.64	10^{-2}	-0.98	10^{-6}
Act79B	structural constituent of cytoskeleton, cytoskeleton organization and biogenesis	-1.07	10^{-3}	-0.77	10^{-5}

Hsp26	determination of adult life span, response to heat	-0.89	10^{-3}	-0.94	10^{-6}
Hsp27	determination of adult life span, response to heat	-1.54	10^{-6}	-1.33	10^{-11}
tld	metalloendopeptidase activity, imaginal disc-derived wing vein morphogenesis, specific transcriptional repressor activity, regulation of transforming growth factor beta receptor signaling pathway, positive regulation of BMP signaling pathway	-0.74	10^{-2}	-0.67	10^{-4}
tok	calcium ion binding, imaginal disc-derived wing vein morphogenesis	-1.20	10^{-4}	-0.78	10^{-5}

Additional file 3.

Representatives of each gene class
