# Supplementary material for: Gene expression following induction of regeneration in Drosophila wing imaginal discs. Expression profile of regenerating wing discs
Source: BMC Dev Biol. 2010 Sep 2;10:94. doi: 10.1186/1471-213X-10-94 (PMC2939566; doi:10.1186/1471-213X-10-94)
Supplement: Additional file 4 — Number of genes on each class in C0→C24 and C24→C72. For each microarray we display the number of misregulated genes distributed in classes according to the gene catalogue. [file 1471-213X-10-94-S4.PDF]

	C0→C24			C24→C72		
	↑	↓	TOTAL	↑	↓	TOTAL
**CLASS I**	**495 (82%)**	**537 (93%)**	**1032 (87%)**	**0 (0%)**	**0 (0%)**	**0 (0%)**
**CLASS II**	**0 (0%)**	**0 (0%)**	**0 (0%)**	**50 (44%)**	**80 (49%)**	**130 (46%)**
**CLASS III**	**48 (8%)**	**2 (0%)**	**50 (4%)**	**2 (0%)**	**48 (29%)**	**50 (18%)**
**CLASS IV**	**64 (10%)**	**37 (7%)**	**101 (9%)**	**64 (56%)**	**37 (22%)**	**101 (36%)**

Additional file 4.

Number of genes on each class in C0→C24 and C24→C72
