# Supplementary figures and images for: Gene expression following induction of regeneration in Drosophila wing imaginal discs. Expression profile of regenerating wing discs
Source: BMC Dev Biol. 2010 Sep 2;10:94. doi: 10.1186/1471-213X-10-94 (PMC2939566; doi:10.1186/1471-213X-10-94)

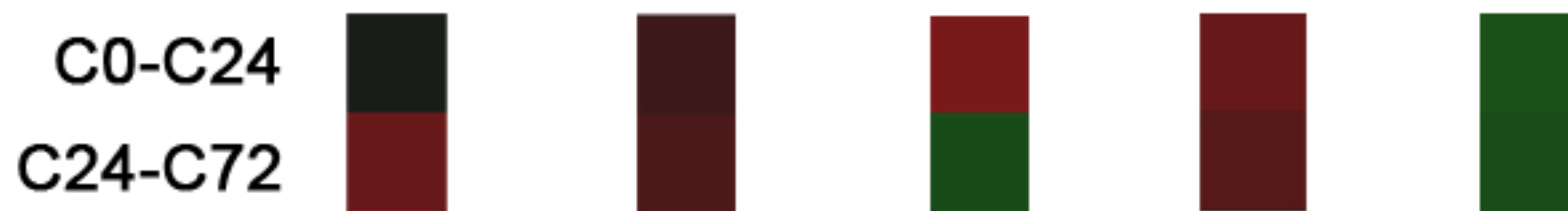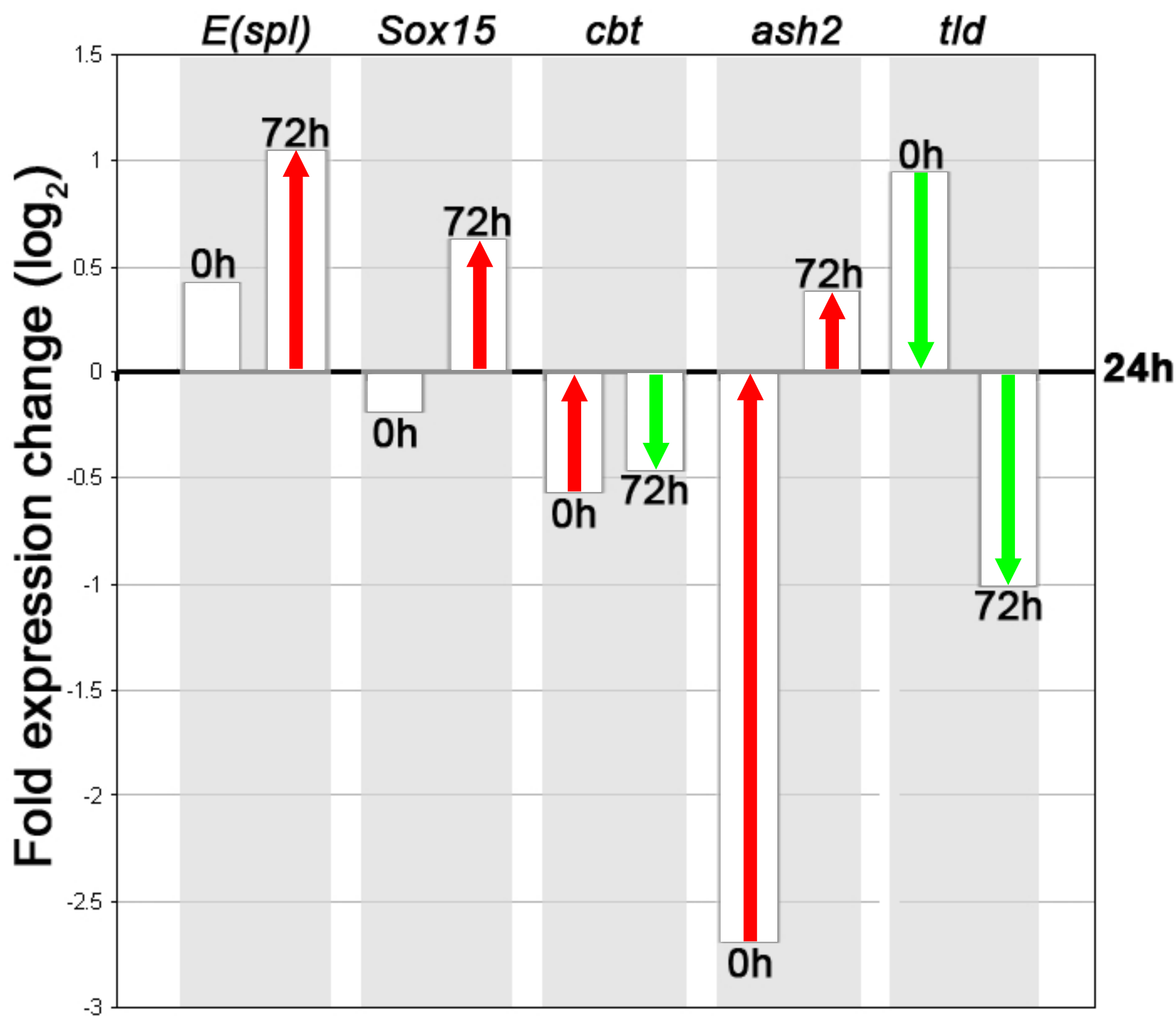

Supplement: Additional file 5 — Quantitative RT-PCR for target genes in regenerating discs after 0, 24 and 72 hours. Twenty-four hour expression levels were used as reference for comparison (baseline). Arrows represent significant expression changes (P < 0.005); red arrows indicate upregulation and green arrows downregulation. [file 1471-213X-10-94-S5.PDF]

# CLASSIII

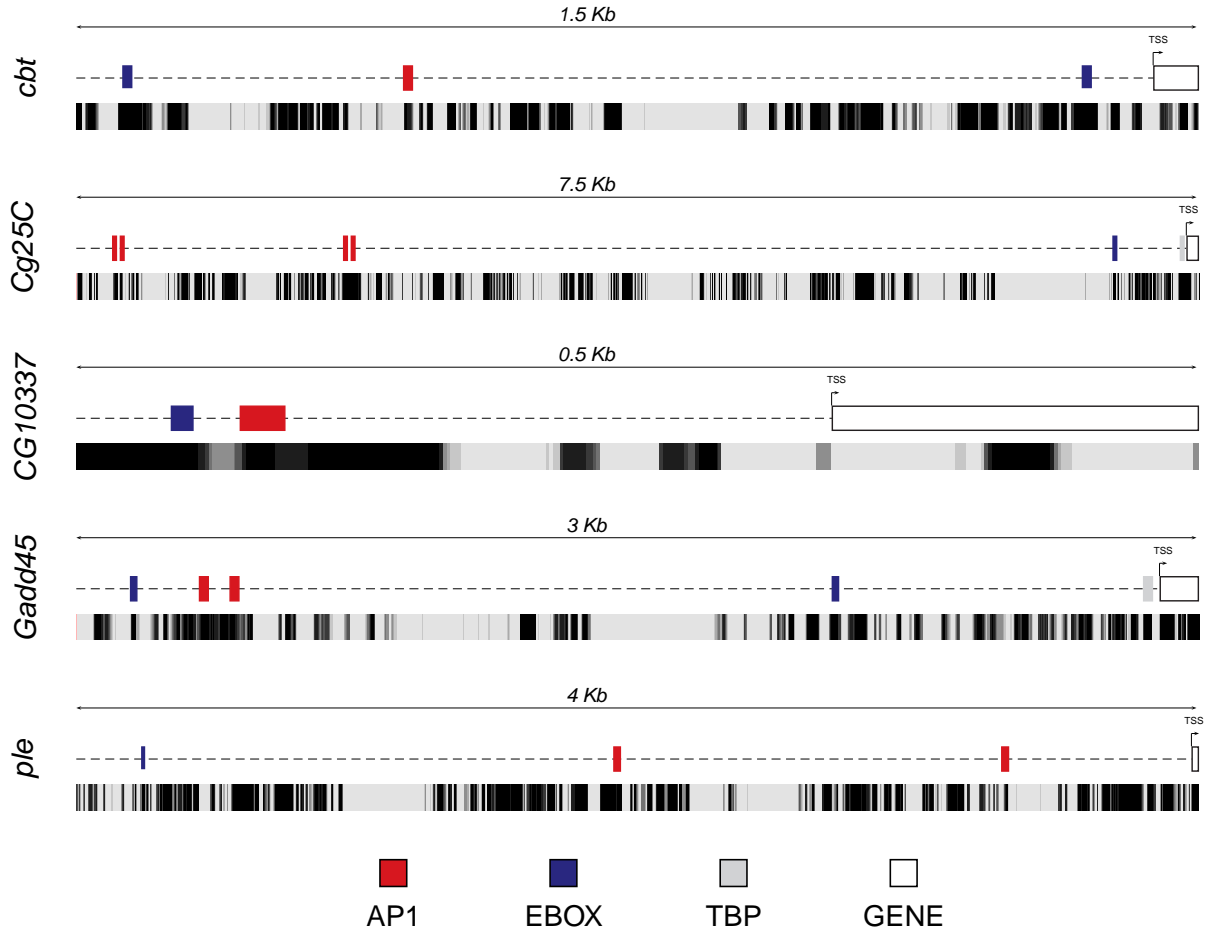

Supplement: Additional file 6 — AP1 sites and E-boxes identified in the promoter region of Class III genes. For each gene we display the length of the promoter sequence, the position of AP1 sites (in red) and E-boxes (in blue), the beginning of the gene (in white) and the conservation level of the sequence according to the multiple alignment of Drosophila species (UCSC genome browser Conservation track). [file 1471-213X-10-94-S6.PDF]
